# Plasma biomarker proteins for detection of human growth hormone administration in athletes

**DOI:** 10.1038/s41598-017-09968-7

**Published:** 2017-08-30

**Authors:** Sock-Hwee Tan, Albert Lee, Dana Pascovici, Natasha Care, Vita Birzniece, Ken Ho, Mark P. Molloy, Alamgir Khan

**Affiliations:** 1 Australian Proteome Analysis Facility (APAF), Level 4, Building F7B, Research Park Drive, Macquarie University, Sydney, NSW 2109 Australia; 2 Garvan Institute of Medical Research, New South Wales, NSW 2010 Australia; 3 Present Address: Cardiovascular Research Institute, Yong Loo Lin School of Medicine, National University of Singapore, Singapore, 119077 Singapore; 4 Present Address: Department of Biomedical Sciences, Faculty of Medicine and Health Sciences, Macquarie University, New South Wales, NSW 2109 Australia; 5 Present Address: School of Medicine, Western Sydney University, New South Wales, NSW 2751 Australia; 6 Princess Alexandra Hospital, University of Queensland, Brisbane, QLD 4102 Australia

## Abstract

Human growth hormone (GH) is a naturally occurring hormone secreted by the pituitary gland with anabolic and growth-promoting activities. Since an increased availability of recombinant GH (rGH) for the treatment of GH-deficient patients, GH has been abused in sports and it is prohibited. “GH-isoform” and “biomarkers” tests are currently available for detection of GH abuse in sports, however both methods suffer from shortcomings. Here, we report on a proteomic approach to search for novel protein biomarkers associated with rGH administration in non-elite athletes. In this study, participants received either placebo or rGH for 8 weeks, and were followed over a 6-week washout period. We used 2-D DIGE and iTRAQ LC-MS/MS analyses to expose rGH-dependent marker proteins. Eight rGH-dependent plasma proteins namely apolipoproptein-L1, alpha-HS-glycoprotein, vitamin D-binding protein, afamin, insulin-like growth factor-binding protein-3, insulin-like growth factor-binding protein-ALS, lumican and extracellular matrix proteins 1 were identified. Apolipoprotein L1 and alpha-HS-glycoprotein were validated by Western blots to confirm their identities and expression patterns in rGH- and placebo-treated subject cohorts. Independent confirmation of these putative GH-responsive biomarkers would be of value for clinical practices and may have sports anti-doping utility.

## Introduction

Human growth hormone (GH) is a naturally occurring, 191 amino acid peptide hormone secreted by the pituitary gland which consists of several isoforms, mainly 22 kDa and 20 kDa isoforms^1^. There are splice variants of pituitary GH^2^, and proteolytic fragments have been reported^3^. In addition to various isoforms, post-translational modifications of the pituitary GH such as deamidation, acetylation, and phosphorylation have been reported^2^. Owing largely to its anabolic, lipolytic and growth promoting properties, there is a widespread misuse of GH as a performance enhancer in competitive sports^4^. The recombinant form of human growth hormone (rGH) produced in *Escherichia coli* bacteria principally for the clinical treatment of endocrine disorders is a 22 kDa protein that has identical amino acid sequence with the principle pituitary GH isoform. Consequently, analytical methods to detect exogenous GH misuse in sports remains extremely challenging^5^.

The World Anti-Doping Agency (WADA) prohibits the use of any form of exogenous GH and growth factor products in sports^6^. GH is listed in the prohibited list under class S2 of peptide hormones, growth factors and related substances and mimetics. Although the International Olympic Committee (IOC) has banned the use of exogenous GH in sports since 1989, followed by WADA listing GH as a prohibited substances in 1999^6, 7^, an adverse finding of GH abuse was only identified for the first time in 2010^8^.

Currently, there are two approaches used by the WADA-accredited laboratories for detection of GH abuse in sports: **A**) GH isoform test^9^, and **B**) GH biomarkers test^10^. The isoform test is based on two immunoassays that distinguish between the 22 kDa GH isoform and all other endogenous GH isoforms using specific monoclonal antibodies (MAbs)^11^. Administration of exogenous GH increases the concentration of the 22 kDa GH isoform only, disrupting the ratio of 22 kDa isoform to all pituitary isoforms, which allows identification of GH abuse in athletes. Although this direct isoform detection method is technically robust, it has some inherent limitations, such as: i) the time window for detection is very narrow, up to 36 h after the last injection depending on the administered dosage concentration and athlete gender^12^, and ii) cannot detect commercially available purified pituitary derived GH administration^5, 12^.

The Biomarkers test is an indirect method which is based on measuring increased levels of GH-responsive proteins such as insulin-like growth factor 1 (IGF-1) and pro-collagen type III amino-terminal pro-peptide (P-III-NP)^10, 13, 14^. However, there were some concerns that ethnicity and potential effects of sport injuries may alter the production rate of biomarkers^7, 15^. Later study confirmed both biomarkers (IGF-1 and P-III-NP) varied minutely between ethnicity, whereas sport injury did not significantly affect the biomarkers test^7^. A major advantage of using the biomarkers test is the wider time window of detection compared to the isoform test^5^. The concentration of IGF-1 and P-III-NP markers progress at different rates; the former generally increases to its maximum within two weeks following GH injection, while the latter progresses gradually and usually peaks within 4–6 weeks^16^. After cessation of GH administration, IGF-1 levels decrease rapidly within a week to homeostatic levels whereas P-III-NP decline more slowly, returning to baseline by six weeks^16^. This provides an opportunity to use the biomarkers test for both ‘in and out of competition’ stages.

Despite having an advantage of a wider time window for detection, the biomarker test contains some weaknesses, such as concentration of IGF-1 in circulation is largely age and gender dependent^14^ and partially sport and exercise dependent^13, 17^. To address this variability, several criteria (age, gender, sport, ethnicity etc.) need to be taken into consideration in order to identify exogenous GH administration accurately. This variability has contributed at least in part, for the limited number of positive GH abuse cases in sports to-date.

In this study, we took an unbiased proteomic screening approach aimed to find novel biomarker proteins of GH administration in athletes. New or different analytical techniques or approach are likely to identify novel biomarkers^2, 5, 18, 19^; hence, we have employed 2-D DIGE and iTRAQ LC-MS/MS proteomic approaches to search for novel GH biomarker which may strengthen the current biomarker test.

## Results

### Immunodepletion and 2-D DIGE analysis of plasma proteins

**Figure 1:**
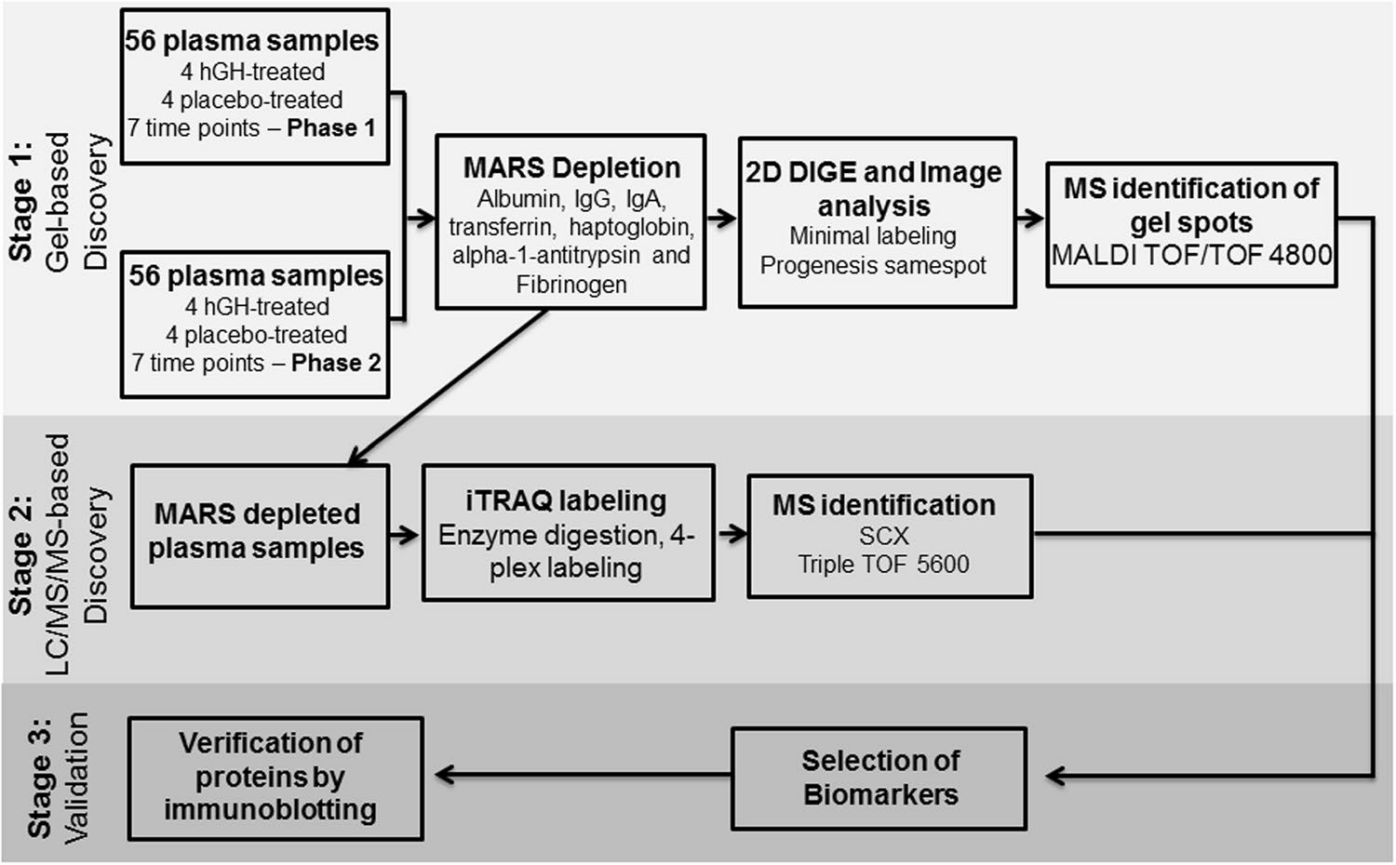
Experimental workflow. Seven high-abundance proteins (MARS-Hu7 column) were immunodepleted in plasma samples. Low abundance proteins were used for further analysis while the high abundance proteins were discarded. **Stage 1:** Depleted plasma samples were labeled with minimal CyDyes and separated on 2D gels in two phases. Images were compared and analyzed using SameSpot software and differentially expressed spots were identified by MALDI MS/MS analysis. **Stage 2:** Depleted plasma samples were digested with trypsin enzyme and labeled with 4-plex iTRAQ reagents. Labeled peptides were separated by SCX and identified using Triple TOF 5600. **Stage 3:** Biomarkers were selected from gel-based and MS-based discoveries and selected two biomarkers were validated using 1D and 2D Western blots.

Plasma samples obtained from non-elite athletes whom were administrated with 2 mg/day rGH or placebo for 8 weeks^4^ was analysed by 2-D DIGE following immunodepletion of the top seven most abundant plasma proteins (Fig. 1). The samples used in this study contained increased levels of IGF-1^4^, a known GH biomarker.

**Figure 2:**
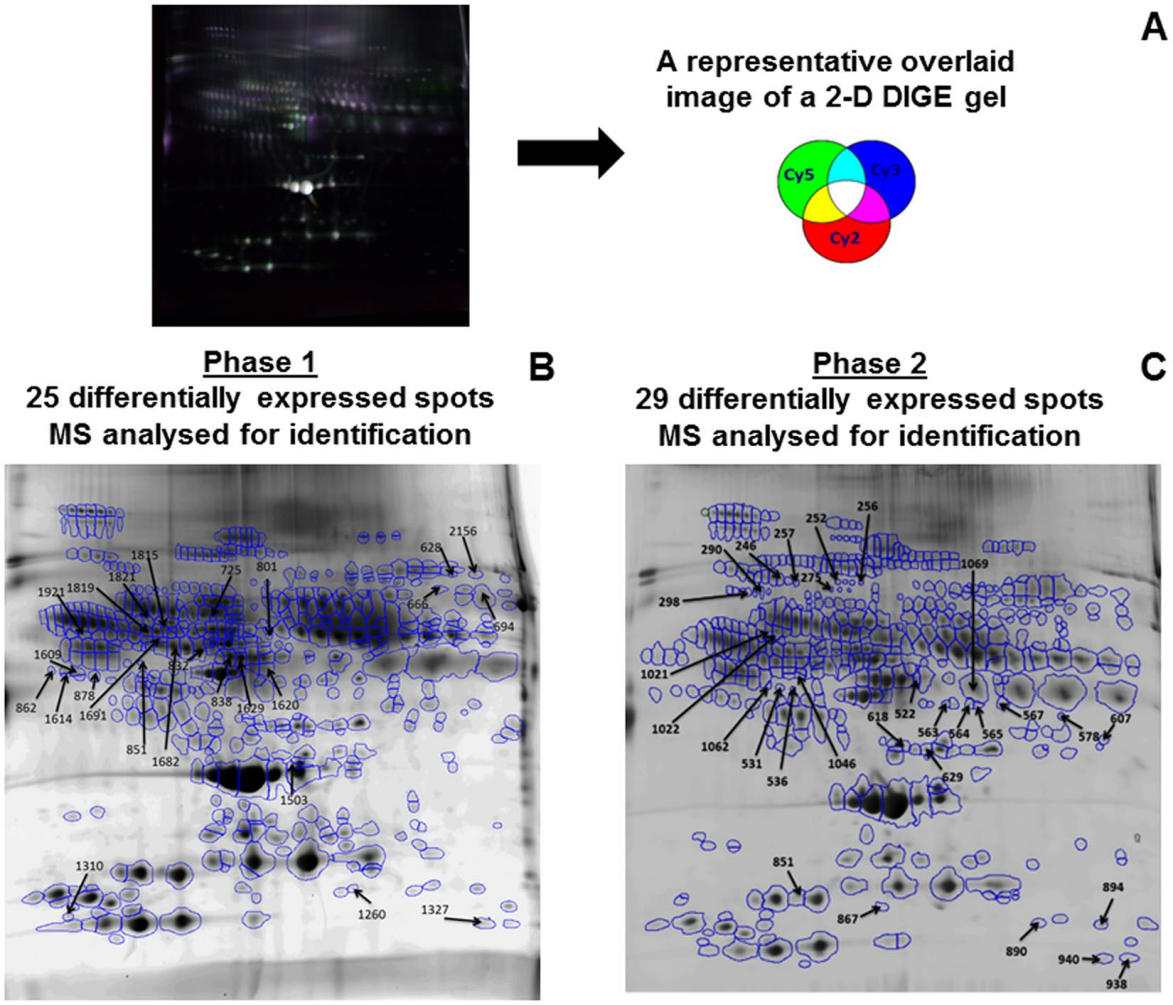
A representative 2-D DIGE gel image (**A**), protein spot detected by SameSpot software followed by statistical analysis (**B** and **C**), and MALDI MS/MS analysis of differentially expressed spots after the second normalization (arrowed spots) for protein identification (**B** and **C**).

Analysis of the chromatogram of a control plasma sample injected prior to and at the completion of each phase (180 injections later) demonstrated excellent reproducibility indicating that no large bias was introduced from the immunodepletion process (Supplementary Information Fig. S1A). We estimated that ~40–50% of the total plasma proteins (by weight) were removed by this process, with the coefficient of variation (CV) determined to be 3.5% from triplicate depletion of a sample (Supplementary Information Fig. S1B). A representative 2-D DIGE image shown in Fig. 2A demonstrates that HAP depleted plasma samples were well resolved. All 56 2-D DIGE images are shown in Supplementary Information Fig. S2.

### Gel image analysis for identification of differentially expressed protein spots

**Figure 3:**
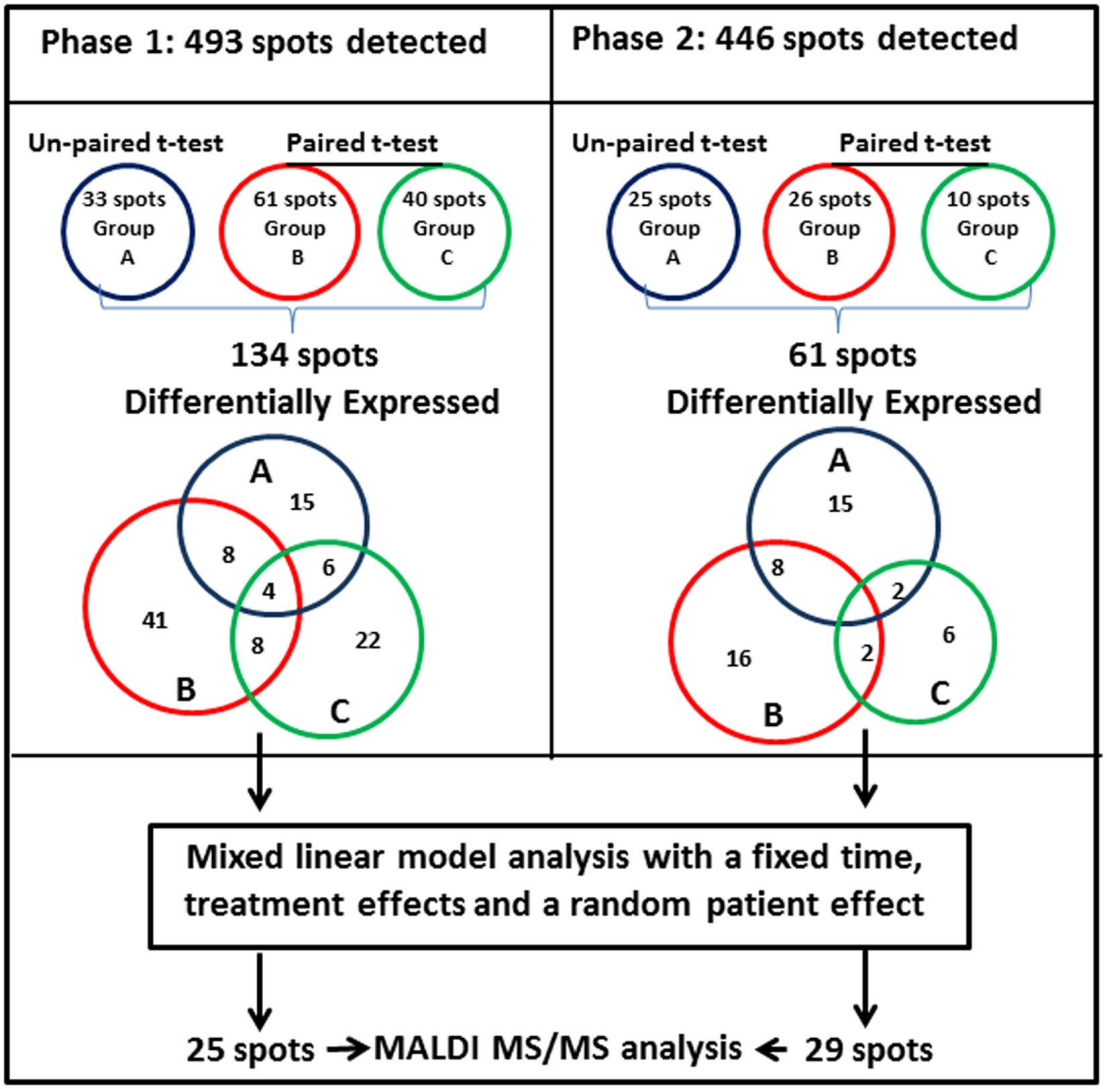
Venn diagram showing differentially expressed spot numbers detected on 2-D DIGE gels in both Phases 1 and 2 (fold-change > 1.5 and *p* < 0.05). **Group A** = differentially expressed between rGH-treated and placebo-treated; **group B** = within rGH-treated; **group C** = within placebo-treated. In A, B, and C groups data from all the sample collection time points were considered.

A total of 112 individual plasma samples were divided into two equal groups of 56 samples for analysis by 2-D DIGE in two Phases (Fig. 1). Each gel contained a pooled reference sample to facilitate gel alignment and matching. After manual editing of gel spot boundaries, Phase 1 analysis revealed 493 spots, while analysis of Phase 2 detected 446 spots. (Figs 2B,C and 3). Using the pooled reference sample in each gel (all 28 gels in Phase 1), we estimated the coefficient of variation (CV) between gels at 8% based on total number of auto-detected spots. This demonstrated an excellent quantitative reproducibility of 2-D DIGE gels.

Normalised spot volumes were measured and any spot volume differences +/−1.5-fold were analysed by pairwise comparisons. Pairwise comparisons were made in three groups; unpaired student t-test was performed for comparison between rGH-treated and placebo-treated groups (**Group A**) whilst paired student t-test was performed to compare temporal changes within the rGH-treated (**Group B**) and temporal changes within the placebo-treated group (**Group C**). For group A, differences were examined at ‘each’ time point separately, to identify ‘any’ time dependence. In Phase 1 analysis, 134 spots were found to be differentially regulated for comparisons amongst groups A, B and C, while 61 spots were changed in Phase 2 analysis (Fig. 3). There were some unique and common spots between the three goups in both phases. The variation in numbers of differentially expressed spots in Phase 1 and Phase 2 is commonly experienced with 2D gel image analysis^20^ and can be attributed to inter-individual subject variation, as each Phase examined different individuals. The 134 and 61 differentially expressed spots were then further analysed by a mixed linear model with a fixed time and treatment effects and a random patient effect. The criteria used for selection of candidate spots were (a) mixed model p-value < 0.05 for either the time effect, treatment effect or their interaction, and (b) relative fold-change in the rGH-treated samples along the time course >50% OR between rGH-treated and placebo-treated samples >50%, and (c) relative fold-change in the placebo-treated samples along the time course <30% (described in detail in Supplementary Information page 9). This led to the selection of 25 spots from Phase 1 and 29 spots from Phase 2 which were then subjected to MALDI-MS/MS for identification (Figs 2B,C and 3).

### MALDI-TOF/TOF analysis of differentially expressed spots

A total of 54 spots in Phases 1 and 2 were selected, 52 spots were excised for identification by MALDI-MS/MS analysis. Two spots were not visible either in the re-stained DIGE gels or in preparative gels, and therefore could not be excised for MS analysis. Out of 52 spots analysed by MS, 39 spots were identified as follows: from the Phase 1 analysis, 20 differentially expressed spots matched to 12 proteins (Supplementary Information Table S1). These included: alpha-1-antitrypsin (AAT), alpha-2-HS-glycoprotein (AHSG), angiotensinogen (AGT), apolipoprotein L1 (APOL1), apolipoprotein A-1 (APOA-1), complement C3 (C3), kininogen-1 (KNG1), leucine-rich alpha-2-glycoprotein (LRG), serum amyloid A-1 protein (SAA1), serum amyloid A-2 protein (SAA2), vitamin D-binding protein (VDBP) and vitronectin (VN). From the Phase 2, 19 differentially expressed spots matched to 13 proteins (Supplementary Information Table S1). These included: alpha-1-antichymotrypsin (ACT), AHSG, anti-thrombin-III (ATIII), apolipoprotein E (APOE), apolipoprotein L1 (APOL1), beta-2-glycoprotein 1 (β2GP1), complement C1s subcomponent (C1s), complement C4-A (C4A), complement C4-B (C4B), inter-alpha-trypsin inhibitor heavy chain H4 (ITIH4), (KNG1), VDBP and VN.

Among the identified proteins, five proteins namely; APOL1, AHSG, VDBP, VN, and KGN1 were shown to be differentially expressed in rGH-treated group from both analysis Phases (Supplementary Information Table S1). Further evaluation showed that both KNG-1 and VN demonstrated different expression patterns between Phases 1 and 2 so were excluded from further evaluation. We suspect this is due to variability amongst individuals analysed in the two separate Phases. We also identified apolipoprotein A-1, alpha-1 antitrypsin, and inter-alpha trypsin inhibitor heavy chain H4 proteins as differentially expressed in one of the analysis Phases only, so, were not evaluated further. Interestingly, previous studies have reported these three proteins to be differentially expressed in rGH or CJC-1295 (an analogue of growth hormone releasing hormone), administered subjects, so while they were not followed further by us, some concordance was observed^21, 22^.

### iTRAQ LC MS/MS analysis

**Figure 4:**
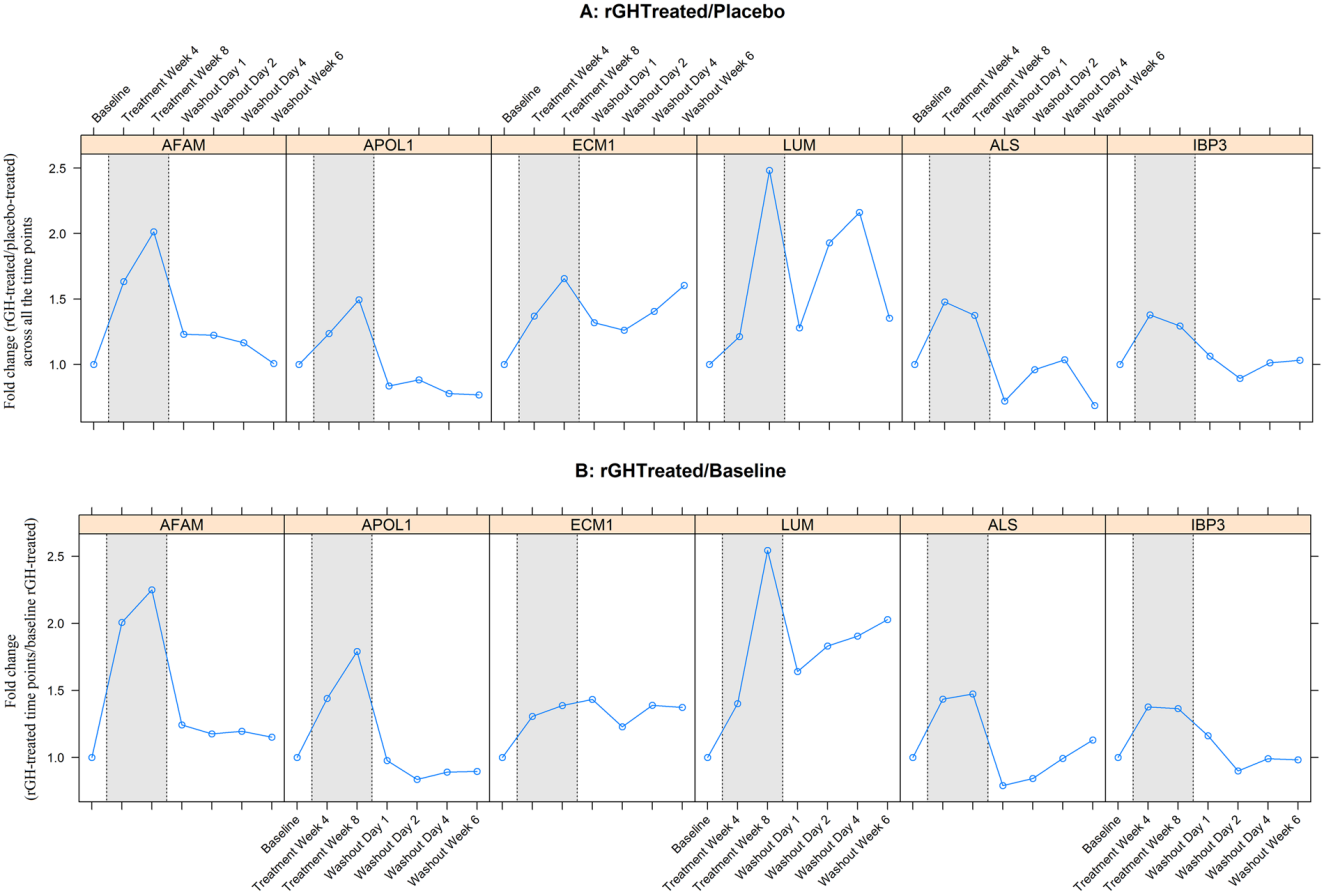
rGH responsive plasma proteins identified and quantified by iTRAQ LC-MS/MS analysis from pooled samples. (**A**) rGH-treated vs placebo-treated at base line, treatment week 4, treatment week 8, washout at day 1, day 2, day 4, and at 6 weeks after the last rGH injection. (**B**) rGH treated/treated at baseline at treatment week 4, treatment week 8, washout at day 1, day 2, day 4, and at 6 weeks after the last rGH injection. In both Figs (**A** and **B**), (a) two proteins Insulin-like growth factor-binding protein complex acid labile subunit (IGFBP-ALS) and Insulin-like growth factor-binding protein 3 (IBP3) were previously reported by other groups as rGH plasma biomarkers; (b) four novel rGH plasma biomarkers Afamin (AFAM), Apolipoprotein L1 (APOL 1), Extracellular matrix protein 1 (ECM1), and Lumican (LUM) identified by iTRAQ LC-MS/MS analysis in this work.

As an independent approach to 2-D DIGE we used iTRAQ-LC-M/MS for peptide detection and quantification (experimental design and search results in Supplementary Dataset). It is important to note the following differences between these approaches. Unlike 2D-DIGE which considers proteoforms as independent gel spots, iTRAQ protein quantitation is inferred from the detected tryptic peptides. Additionally, for iTRAQ analysis, samples for given time points and groups were pooled due to limited sample availability, whereas in the 2D-DIGE experiments each sample was independently assessed. 385 proteins were identified from six iTRAQ LC-MS/MS runs of HAP immunodepleted plasma. 101 proteins were differentially expressed in **Group A** comparison (i.e. differentially expressed between rGH-treated vs placebo-treated) and 93 proteins in **Group B** comparison (i.e. differentially expressed within rGH-treated, baseline vs other time points) based on +/− 1.2-fold change (p ≤ 0.05) during rGH administration period. These differentially expressed proteins were filtered following ≥2 peptides match and 95% confidence level in the Paragon search algorithm, and 7 unique proteins were identified in **Group A** and 10 unique proteins in **Group B** (top scored proteins in Supplementary Dataset). Six proteins that were consistently differentially expressed in both group comparisons were APOL1, Insulin-like growth factor-binding protein complex acid labile subunit (IGFBP-ALS), afamin, insulin-like growth factor-binding protein 3 (IGFBP 3), lumican, and extracellular matrix protein 1 (Fig. 4 and Supplementary Dataset). Amongst these proteins, APOL1 was also identified as differential in the 2-D DIGE analyses, while the remaining proteins were not shown to be differential from the 2-D DIGE analyses.

### Candidate rGH plasma biomarkers

**Figure 5:**
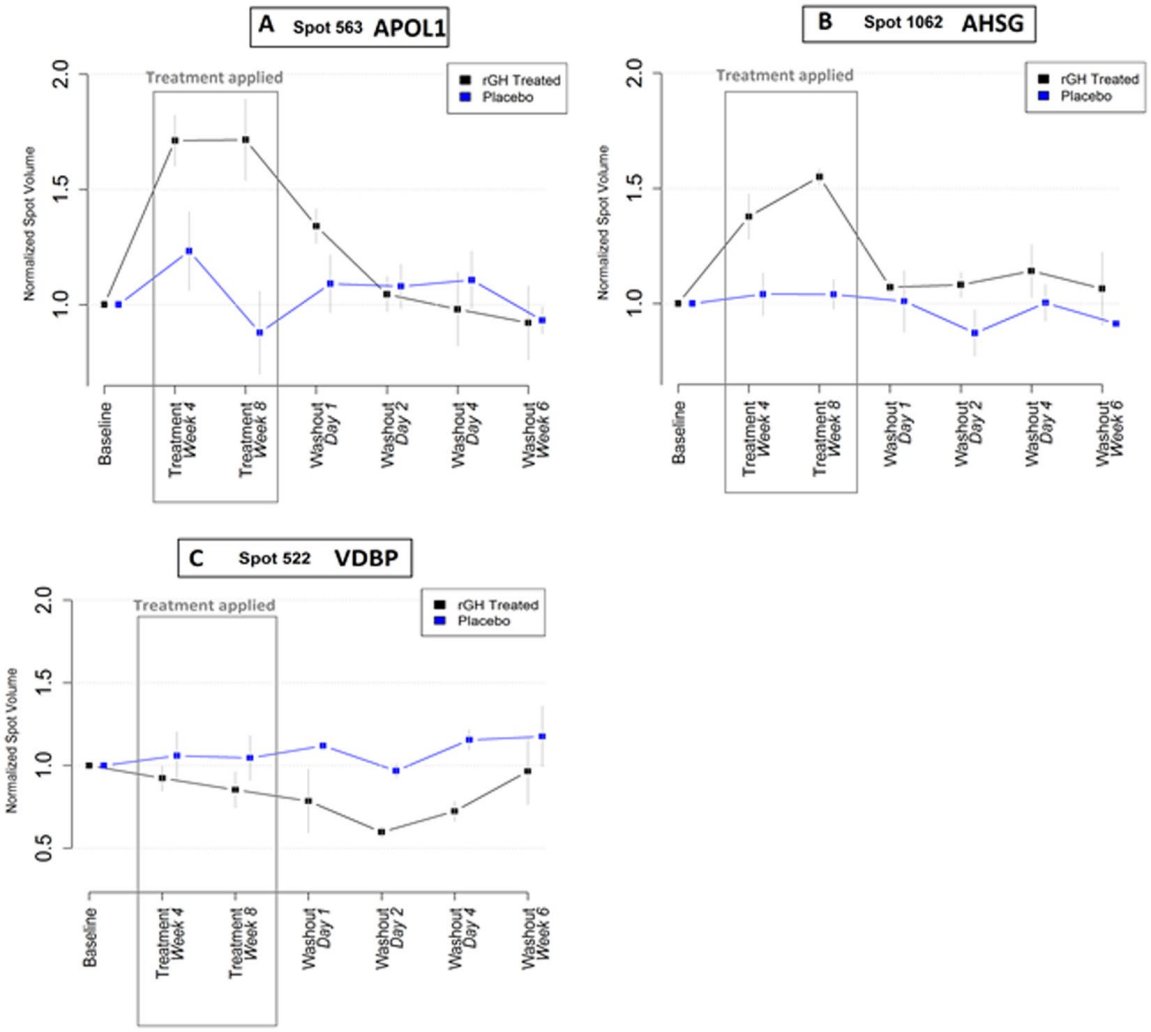
Differential expression of three spots corresponding to (**A**) APOL1, (**B**) AHSG, and (**C**) VDBP based on DIGE gel image analysis. Both APOL1 and AHSG were up-regulated and VDBP was down-regulated during the rGH treatment period (boxed area). Higher concentrations of APOL1 and AHSG observed during rGH administration period which were gradually become normal during the washout period. Concentration of VDBP was decreased during rGH administration and maintained lower concentration until the end of washout period.

APOL1, AHSG and VDBP were selected based on 2-D DIGE analysis (Table 1, Fig. 5) and APOL1, IGFBP-ALS, afamin, IGFBP 3, lumican and ECM1 were selected based on iTRAQ LC-MS/MS analysis as rGH biomarkers (Table 1, Fig. 4). APOL1 was differentially expressed (up-regulated) and identified by t-test in both phases, 2-way ANOVA, and quantitative iTRAQ MS/MS analyses (Table 1). AHSG was differentially expressed (up-regulated) and identified by t-test analysis in the gel work in both phases and also 2-way ANOVA analysis (Table 1). VDBP was differentially expressed (down-regulated) in the gel analyses in both phases. However, two-way ANOVA showed that VDBP was not statistically significant (Table 1). Quantitative iTRAQ MS/MS analysis also identified AHSG and VDBP peptides but the expression differences of both proteins between rGH-treated and placebo-treated individuals was not confirmed as significant using this method of analysis. Afamin, IGFBP3, IGFBP-ALS, lumican and ECM1 proteins were significantly differentially expressed and identified from quantitative iTRAQ MS/MS experiments (Table 1, Fig. 4).

**Table 1:** Selection of rGH biomarkers from differentially expressed^◊^ proteins^ł^ identified by multiple techniques.

Name of the biomarkers	Change in rGh-treated	2-D gels & T-test	2-way ANOVA	Quantitative iTRAQ MS/MS	Quantitative Western blot
APOL1	↑	√	√	√ **	√
AHSG	↑	√	√	NS	√
VDBP	↓	√	NS	NS	NT
Afamin	↑	NI	NT	√ **	NT
IGFBP-3	↑	NI	NT	√ *	NT
IGFBPC-ALS	↑	NI	NT	√ *	NT
Lumican	↑	NI	NT	√ *	NT
Extracellular matrix	↑	NI	NT	√ *	NT
protein 1					

Note: **◊** = based on T-tests (*P* ≤ 0.05); ł = each protein had several isoforms on 2-D DIGE gels; NI = Not Identified; NT = Not Tested; NS = protein identified but not significantly different; **Fold change > 1.5 (*P* =  < 0.05); *Fold change > 1.2 but < 1.5 (*P* = < 0.05); ↑ = up-regulated; ↓ = down-regulated. Eight rGH plasma biomarkers of which six novels such as apolipoproptein-L1 (APOL1), alpha-HS-glycoprotein (AHSG), vitamin D-binding protein (VDBP), afamin, lumican and extracellular matrix protein −1 (ECM-1) and two previously reported biomarkers such as insulin-like growth factor-binding protein-3 (IGFBP-3) and insulin-like growth factor-binding protein-ALS (IGFBPC-ALS) were identified by gel-based and LC-MS/MS-analyses in this work.

### Western blot analysis for validation of rGH plasma biomarkers

We selected two candidate biomarker proteins that have not previously been shown to be GH-responsive. Both APOL1 and AHSG were consistently shown to be GH-responsive from our 2-D DIGE (Fig. 5) and this trend was also observed for APOL1 from the iTRAQ data (Fig. 4). However, AHSG did not differ significantly between rGH-treated and placebo-treated samples in iTRAQ MS/MS analaysis. We carried out 1-D SDS PAGE and Western blotting of samples from all the placebo control and rGH treated subjects collected at all seven time points for both APOL1 and AHSG. Further, using a subset of samples from each treatment group, we conducted 2-D Western blot analysis. A subset of samples randomly selected based on the availability of remaining plasma. 2-D Western blotting was carried out to: i) observe the expressional differences in various isoforms, and ii) correlate the location of the proteins by western blotting with the results obtained by 2D DIGE gels.

#### APOL1

**Figure 6:**
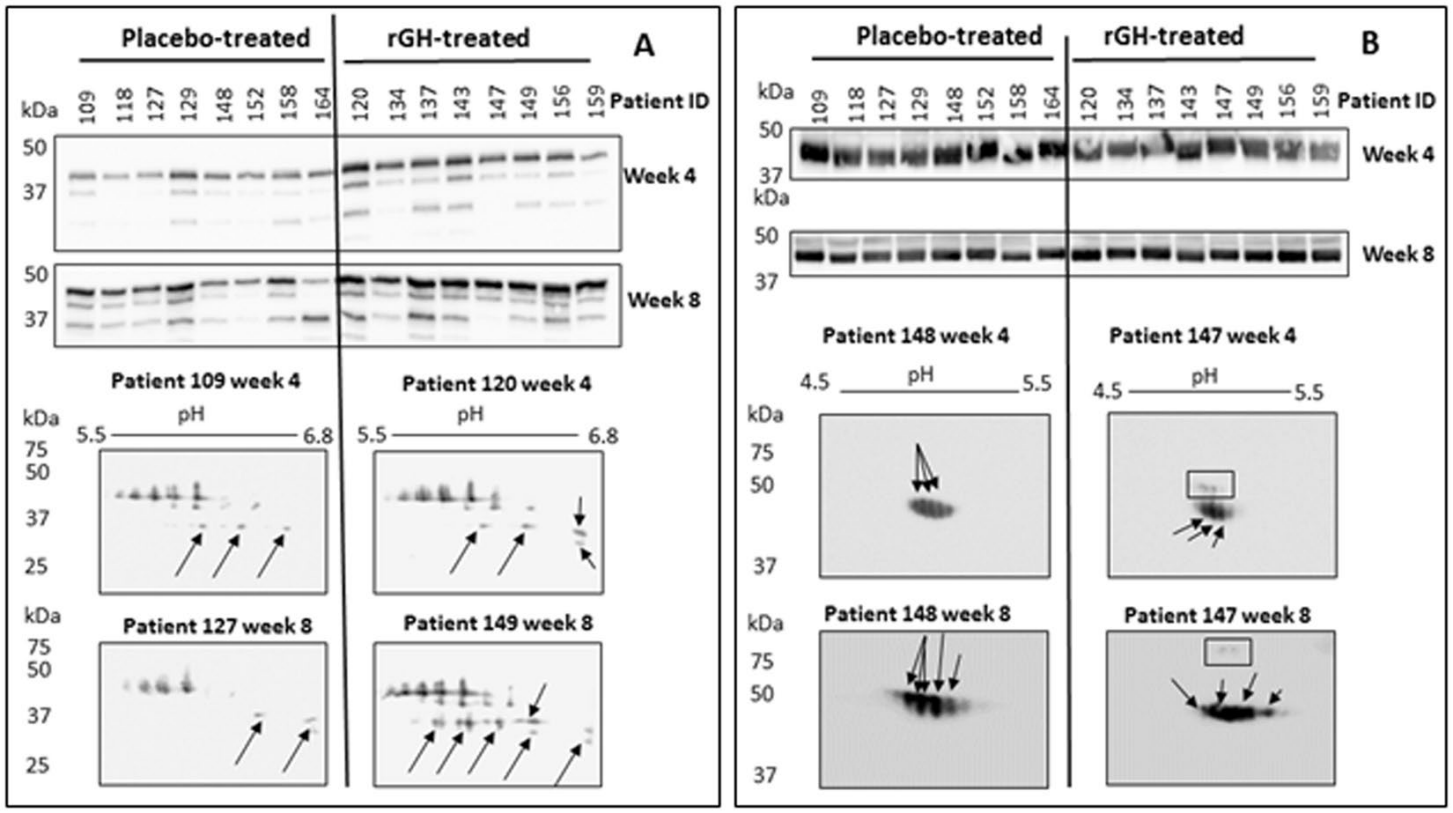
Western blot analysis of two rGH plasma biomarkers. (**A**)** = **APOL1 and (**B**) AHSG. For 1-D Western blot analysis, all the samples were analysed however, data presented in this figure only for treatment weeks (4 and 8). For 2-D Western analysis, different subject’s samples were analysed due to limited sample availability. (**A**) It appeared more APOL1 isoforms and over-expressed in the lower ~37 kDa region in patients treated with rGH compared to placebo-treated patients as indicated by the arrows. (**B**) It appeared that AHSG has ‘train’ of isoforms ~45 kDa in the patients treated with placebo whilst two sets of isoforms (one set ~45 kDa and another set of isoforms with higher masses, boxed) were observed in patients treated with rGH only. Western blot images shown in this Figure were cropped; uncropped full length images are shown in the Supplementary Information Fig. S6.

Three isoforms were observed in the 1D Western blot that ranged from approximately 35–45 kDa, and it appeared that these isoforms differed between subjects and also between treatment groups (Fig. 6A). Densitometric analysis of APOL1 comparing the placebo-treated (n = 8) and rGH-treated (n = 8) subject groups determined statistical significance with increased expression of ~2.1- and 1.8-fold at weeks 4 and 8 respectively (Supplementary Information Fig. S3A). The heavier isoform ~40 kDa is likely to be the canonical glycosylated isoform of APOL1; whilst the remaining two isoforms of lower mass may be truncated isoforms associated with splicing or degradation^23^. Densitometric analysis showed that the heavier and lighter isoforms were up-regulated in the rGH treated subjects. We further investigated APOL1 in placebo and rGH-treated samples by 2D western blots as it was apparent from the 1D western blots that differential expression pattern exists amongst the heavier and lighter isoforms. These experiments showed that the 40 kDa isoform is up-regulated in rGH administered subjects based on the intensity and number of the protein spots (Fig. 6A). Additional protein spots corresponding to the ~35 kDa isoform were observed in the rGH treated samples with an iso-electric point between 6.2–6.8 (indicated by arrows in Fig. 6). This suggested that upon rGH treatment, expression of both 40 kDa and 35 kDa isoforms were elevated and maintained until treatment week 8 (Fig. 5). Isoelectric points of these APOL1 isoforms observed on the 2D western blots matched with those obtained from 2D DIGE images analysis; in both cases, they were up-regulated during rGH treatment period and gradually returned to baseline levels during the washout period. Western analyses confirmed the identity of APOL1 spots identified from 2-D DIGE gels.

#### AHSG

1-D Western blot analysis detected a broad band between 35–40 kDa area in all the subjects in both placebo and rGH-treated groups (Fig. 6B). Densitometric analysis of AHSG comparing the placebo-treated (n = 8) and rGH-treated (n = 8) subject groups determined statistical significance with expressional differences of ~0.8-fold and 1.2-fold at weeks 4 and 8 respectively (Supplementary Information Fig. S3B). The band broadness is consistent with protein modification, most likely glycosylation as has been reported previously^24^. In the DIGE gels we identified four AHSG isoforms (spot 1921 in Phase 1 and spots 531, 1021 and 1062 in Phase 2) that were up-regulated particularly at treatment weeks 4 and 8. The spatial locations of gel spots 531 and 1062 (Phase 2) matched with 2-D Western blots however, the molecular weights of spots 1921 (Phase 1) and 1021 (Phase 2) were higher and slightly acidic on DIGE gels compared to the spots detected on the 2-D Western blots. It is noteworthy that AHSG and Kininogen 1 proteins were concurrently identified from spot 1921, and AHSG and Vitronectin proteins were identified from spot 1021. Identification of two proteins from a single 2-D gel spot suggests concurrent migration of two proteins on the gel which may have altered their locations on the 2-D gels.

## Discussion

We applied an unbiased proteomic approach using two independent techniques in a search for protein biomarkers associated with GH administration in non-elite athletes. We used plasma samples from a previously conducted placebo controlled clinical study^4^ which demonstrated an increase in plasma IGF-1, a known rGH plasma response biomarker. APOL1, AHSG and VDBP markers were identified as putative rGH response biomarkers from 2-D DIGE analysis. APOL1 was also independently detected from iTRAQ MS analysis, along with IGFBP-ALS, afamin, IGFBP 3, lumican and ECM1. IGFBP3 and IGFBP-ALS have been previously reported by others as rGH response biomarkers^13^. We used Western blotting to independently confirm the quantitative observations for APOL1 and AHSG.

APOL1 belongs to high-density lipoprotein (HDL) or ‘good cholesterol’ which is expressed in the pancreas, liver and also in many other tissues^25^. It has been reported that GH treatment has positive correlation with HDL expression in both healthy men and women^26^. Additionally, APOL1 has been reported as a biomarker for renal diseases^27^, associated with autophagy and renal cell carcinoma, chronic kidney disease, and hypertensive nephrosclerosis^28^, heroin-associated nephropathy, and HIV-associated nephropathy^29^. Other apolipoprotein e.g. Apo-A is increased due to GH administration in GH-deficient adults^30^ and in transgenic mice^31^. To the best of our knowledge, association of increased circulating APOL1 due to GH administration in athletes has not been reported.

AHSG, also known as Fetuin-A, is a ~40 kDa protein that is synthesized and secreted by hepatocytes into the plasma and acts as a carrier protein^24^. AHSG is also expressed in the pituitary gland^32^. It has been shown that AHSG inhibits the action of Leukemia inhibitory factor (LIF) in pituitary corticotropes^33^. As overexpression of LIF leads to expansion of pituitary corticotorpes and suppression of somatotropes, and LIF inhibits GH secretion^34, 35^, such inhibitory effect of AHSG on LIF may result in stimulation of GH secretion. Therefore, our discovery of the GH-induced increase in AHSG invites to investigate whether AHSG plays a role in positive feedback of GH secretion. Among other functions, AHSG is thought to promote endocytosis, fatty acid binding and possesses opsonic properties influencing the mineral phase of bone^36, 37^. AHSG has been also recognized as antagonist of insulin receptor tyrosine kinase activity and is implicated in insulin resistance development^38^. As excess GH induces insulin resistance^39^, the increase in AHSG by GH administration may play a role in mediating this effect. Thus, detailed studies are required to investigate the effect of AHSG in growth hormone physiology.

VDBP, a glycosylated alpha-globulin of 58 kDa, is the main carrier of vitamin D metabolites, namely 25-hydroxyvitamin D3 (25OHD3) and 1,25-dihydroxyvitamin D3 (1,25(OH)2D3) in circulation^40^. Previous studies reported that prolonged GH excess in acromegaly associates with increased serum VDBP and GH administration in healthy men elevates circulating VDBP^41, 42^. However, serum VDBP concentrations remain unchanged after two months of GH administration in girls with Tuner Syndrome^43^. In our work, three VDBP isoforms (spots 838 and 1629 in Phase 1 and spot 522 in Phase 2) demonstrated down-regulation in rGH-administered subjects.

Afamin is a member of the albumin gene family and binds to vitamin E^44^. Afamin associates with hyperlipidaemia, metabolic syndrome and insulin resistance^45^. There are no published studies investigating growth hormone effect on afamin. Lumican, an extracellular matrix protein (ECM), and ECM1 promote collagen fibril organisation and tissue repair, and play a key role in the control of growth factor signalling^46^. Abnormal ECM remodelling has been linked to obesity and insulin resistance, and animal studies provide now evidence that insulin resistance after GH administration in mice involves the upregulation of the extracellular matrix in muscle^47^. There are no studies investigating the effect of growth hormone administration on circulating lumican and ECM1 from the available literature.

In a recent report^48^, fibronectin has been described as a potential biomarker for the detection of rGH abuse. We have also identified fibronectin from iTRAQ MS/MS analysis in rGH-treated subjects (Supplementary Dataset) however, it did not satisfy our reporting criteria.

Each of the candidate biomarkers identified in this work were overexpressed during the GH administration period (except VBDP) and their concentrations returned close to the placebo subjects levels after the day 1 washout period. Unfortunately, such a short post-administration detection window limits the utility of these findings for anti-doping purposes. VDBP was the only candidate biomarker which was repressed due to rGH administration. The concentration of VDBP gradually decreased during rGH administration and stayed low until the end of washout period. Anti-doping laboratories use elevated levels of two biomarkers IGF-1 and type III pro-collagen (P-III-P) for testing athletes for exposure to rGH administration^10^. In this scenario, decreasing VDBP levels offers a different measurement vector that could help strengthen the current biomarker test. While we could not independently confirm the change in VDBP and other candidate biomarkers in this study due to sample availability, such findings from the proteomics analysis offers new knowledge regarding the GH-responsive markers in athletes.

## Methods

### rGH administration in non-elite athletes and blood sample collection

Plasma samples obtained from a previously conducted placebo controlled clinical study^4^ were used in this work. St Vincent’s hospital collected all the samples under Human Research Ethics Committee (HREC) approved protocol (Ref: H03/116) where ethical guidelines were followed to protect subject’s confidentiality and safety. Written consents from all the subjects were obtained that samples may be analysed by the Garvan Institute of Medical Research or its collaborators for future research into developing doping test in sport. rGH and matching placebo were administered in healthy young men and women (non-elite athletes) and the dose of rGH was 2 mg per day, administered as subcutaneous injections over 8 weeks followed by a 6-week washout period. Plasma samples were collected at seven time points: at baseline, two treatment time points (at 4 weeks and at 8 weeks), and after stopping GH or placebo administration (washout day 1, day 2, day 4, week 6). Plasma samples were aliquoted and stored at −80 °C. Prior to proceeding with analysis, we have tested by 2-D gel electrophoresis whether plasma samples used in this work retained integrity after a long period of storage, however, observed similar proteome pattern compared to the freshly collected plasma (Supplementary Information Fig. S4). A total of 112 plasma samples from 16 subjects (8 rGH-treated and 8 placebo-treated) were analysed in two phases using 2-D DIGE analysis. In the first Phase, the first 56 time-point samples from 8 subjects (4 rGH-treated and 4 placebo-treated) and in the second Phase, the corresponding samples from the remaining treatment and placebo groups (Fig. 1). The study was carried out in two independent phases to account for technical variability. We hypothesised that proteins satisfying the selection criteria from independent experiments would provide greater confidence as putative biomarkers.

### Immunodepletion of high abundance proteins from plasma

Seven high abundance plasma proteins (HAPs; albumin, IgG, IgA, transferrin, haptoglobin, antitrypsin, and fibrinogen) were immunodepleted using the Multiple Affinity Removal System (MARS) Human 7 column (4.6 mm × 100 mm) according to the manufacturers’ instructions (Agilent Technologies) using the Agilent Technologies 1260 infinity HPLC system. The flow-through fractions from three consecutive MARS-7 runs of a sample were pooled and precipitated with acetone and protein pellets were solubilized with approximately 200 µl of 2D buffer (7 M urea, 2 M thiourea, 4% (w/v) CHAPS) for DIGE gel analysis. Identical pellets from each of the flow-through fractions was solubilized with 0.25 M triethylammonium bicarbonate (TEAB) and 0.05% (w/v) SDS at pH 8.5 for iTRAQ LC-MS/MS analysis (see below). Total protein concentrations were determined using the Bradford protein assay kit and concentrations were validated by densitometric analysis of 1-D gel bands. Protein concentration was re-adjusted following densitometric analysis as required.

### Labelling of proteins with CyDyes and 2D-DIGE

Plasma samples were labelled with minimal CyDyes (Cy2, Cy3 and Cy5) as instructed by the manufacturer (GE Healthcare) with minor modifications; 400 pmol Cy2 or Cy3 or Cy5 dye was used for labelling 100 µg of proteins instead of the recommended 50 µg. A pool of all 56 plasma samples in each Phase was labelled with Cy2 dye as an internal standard. This internal standard was used for all 28 DIGE gels for comparison in each Phase. Samples were pooled as per DIGE gel (Supplementary Information Table S2), reduced and alkylated as described^49^ and rehydrated onto 17 cm ReadyStrip^TM^ IPG strips (Bio-Rad) with linear pH gradient of 4–7 by passive in-gel rehydration method. Isoelectric focusing (IEF) was carried out on an Ettan IPGphor II instrument (GE Healthcare) until a total of 120 KVh was reached and separated in the 2^nd^ dimension on 8–18%T linear gradient gels (dimension 18cmx20 cm) after equilibration following a standard gel running protocol^49, 50^. The gels were run at 4 mA/gel overnight at 4 °C followed by 40 mA/gel until the tracking dye ran off the gel. All the DIGE gels were scanned immediately using the Typhoon Trio variable mode laser scanner (GE Healthcare) with 100 µm resolution and excitation and emission wavelengths specific to individual CyDye. Following scanning, the gels were stored in fixative solution (10% (v/v) methanol and 7%(v/v) acetic acid) at 4 °C in the dark.

### Preparative 2-D gel electrophoresis for protein identification

Differentially expressed protein spots were either excised directly from the re-stained DIGE gels or preparative gels. Since our robotic spot cutter, equipped with a CCD camera and UV as a source of light, was unable to acquire image of CyDye labelled proteins due to incompatible wavelengths, the DIGE gels were re-stained with SYPRO Ruby for spot cutting. However, due to the sensitivity differences, not all the spots on the DIGE gels were visible on the ExQuest robotic spot cutter (Bio-Rad) after SYPRO Ruby staining. Therefore, preparative gels with higher protein loads (250 µg) were prepared following the same procedures as described for DIGE gels. These gels were stained with SYPRO Ruby and spots were also excised for mass spectrometric analysis. Excised gel spots were digested with up to 200 ng (depending on the number of gel plugs/spot) of trypsin (Promega Biosciences), peptides extracted, desalted and concentrated using zip-tips (perfect pure C18, Eppendorf) as reported^50^.

### Protein identification by MALDI MS analysis

MALDI MS/MS analysis was performed with an Applied Biosystems 4800 *Plus* MALDI TOF/TOF™ Analyzer. The spectra were acquired in reflectron mode in the mass range of 700–4000 Da and were externally calibrated using known peptide standards (bradykinin, neurotensin, angiotensin and ACTH). The instrument was then switched to TOF/TOF mode where the eight most intense peptides from the MS scan were isolated and fragmented. A near point calibration was applied and mass accuracy ≤50 ppm was considered. The peptide peak lists were exported to the Mascot search program (Matrix Science Ltd, version 2.3.2) and searched against *Homo sapiens* in the SwissProt database (2012, 20,246 sequences). Search parameters included peptide mass fingerprinting (PMF) and MS/MS mass tolerances of ± 50 ppm and ± 0.8 Da respectively, and one missed cleavage allowed. For modification of peptides, cysteine alkylation (by acrylamide) and methionine oxidation were considered. When peptide masses were matched to protein sequences in the database, a number of parameters was considered as secondary level search for the top-scored proteins only for identification^50^ such as (i) matched number of peptides; (ii) number of missed cleavage peptides within the matched peptides; (iii) intensities of matched peptides; (iv) number of modified peptides matched; (v) sequence coverage; and (vi) MW and p*I* of the identified protein matched with 2-D gel location.

### iTRAQ 2-D LC-MS/MS analysis

iTRAQ 2-D LC-MS/MS work was carried using a Triple TOF 5600 MS (AB Sciex) coupled with an ultra nanoLC system (Eksigent Technologies). For the top seven HAPs depleted plasma samples, iTRAQ experiment procedures including enzyme digestion, iTRAQ 4-plex labelling and MS/MS data acquisition were the same as reported^51, 52^. Protein quantitation was performed using the Direct Detect Infrared Spectrometer (Merck-Millipore), and 100 µg of proteins from each pooled sample was analysed (eight subjects within each treatment group and within each sample collection time point were pooled as one sample). Each pooled sample was reduced with 5 mM tris(2-carboxyethyl)phosphine (TCEP) for 1 h at 60 °C, alkylated with 10 mM methyl methanethiosulfonate (MMTS) for 10 min at room temperature and then digested with 4 µg of trypsin overnight at 37 °C. The digested samples were labelled for six iTRAQ runs according to the experimental design in Supplementary Dataset. TTP0 (baseline sample in rGH-treated group) was used as the control that each sample was normalised within each run and across all six iTRAQ runs. Labelled-tryptic peptides were combined in an equal ratio and subjected to LC-MS/MS analysis for protein identification and quantitation. ProteinPilot V4.2b (AB Sciex) was used for data processing including peak picking and quantitation analysis with default parameters, and searched using the SwissProt human database (2012, 20,246 entries). The search parameters were as follows: sample type: iTRAQ 4-plex (peptide labelled); cys alkylation: MMTS; digestion: trypsin; instrument type: TripleTOF 5600; special factors: none; ID focus: allow biological modifications. Bias correction was selected.

### Western blot analysis for validation of plasma biomarkers

1-DE and 2-DE Western blot analyses were carried out to validate the two candidate GH plasma marker proteins: APOL1 and AHSG. For 1-DE, 20 µg of neat plasma proteins was resuspended in 4x LDS buffer containing 10 mM DTT, heated at 95 °C for 10 min, and separated on 8–16% Criterion Tris-HCl pre-cast gels (Bio-Rad). 2-DE separation was carried out as described above using 11 cm long IPG strips in the first dimension and 8–16% Criterion Tris-HCl pre-cast gels in the second dimension. 1-DE and 2-DE separated proteins were transferred onto pre-equilibrated PVDF membranes using the Bio-Rad Turbo Transfer apparatus (25 V, 2.5 A for 10 min). Blots were blocked in 3% (w/v) skim milk for 1 h, washed 3x in TBS/T, and incubated with primary antibodies (anti-APOL1 and anti-AHSG [LifeSpan Biosciences Inc.]) in 3% (w/v) BSA in TBS/T overnight at 4 °C. After incubation, membranes were washed 3x with TBS/T, followed by incubation with Goat Anti-Rabbit IgG-HRP for 1 h. Blots were incubated for 5 min in ECL reagent (Merck-Millipore) and imaged using a FujiFilm LAS 3000 CCD camera (Japan). Densitometry analysis was conducted using ImageJ software (v1.47; National Institute of Health).

### Statistical Analyses

For gel image analysis: raw gel images were uploaded into Progenesis “SameSpots” software (Nonlinear Dynamics. UK) and an automated spot detection method was performed. Three different experimental designs were set in Progenesis for spot detection. One design compared between the placebo and rGH-treated subjects at each sample collection time point (**Group A**), one design compared baseline vs all other time points within rGH-treated group only (**Group B**), and the other design compared baseline vs all other time points within placebo-treated group only (**Group C**). Automatic analysis was performed to detect all the spots in all the experiemnts. Each selected spot was verified and manually edited if necessary. Normalized volumes were used to identify spots that were differentially expressed. A cut-off ratio greater than 1.5-fold was imposed and student t-test (unpaired or paired where appropriate) was used to examine the differences (*P* < 0.05). For selection of potential GH plasma biomarkers from gel analysis: further statistical analysis was carried out first at phase levels and then combined analysis followed by final candidate spot selection. A principal component analysis (PCA) of the log-transformed spot data was performed, as well as hierarchical clustering of log-transformed spot data using correlation based distance and complete linkage (Supplementary Information Fig. S5 and description of criteria for selection of candidate spots). The results of both approaches showed that the data from the same subjects clustered together, thus the inter-individual effects were strong. As a consequence, when looking for time or treatment differences we employed a mixed effects linear model with fixed time and treatment effects, and a random subject effect, ran separately for each spot. For iTRAQ MS/MS analysis: detected protein threshold (unused ProtScore) was set as larger than 1.3 (better than 95% confidence). Differentially expressed proteins were categorised in two groups such as differentially expressed between rGH-treated vs placebo-treated (**Group A**), and differentially expressed within rGH-treated- baseline vs other sample collection time points (**Group B**). For top scored protein identification in each group, the following two criteria were utilised: i) proteins needed to be <0.83-fold or 1.2-fold differentially expressed with statistical significance p ≤ 0.05^52, 53^ during rGH administration period (rGH treatment weeks 4 and 8); and ii) the same proteins identified in all sample collection time points (baseline, rGH treatment weeks 4 and 8, and all washout time points).

## Electronic supplementary material

Supplementary Information

Supplementary Dataset
