# Supplementary material for: Plasma biomarker proteins for detection of human growth hormone administration in athletes
Source: Sci Rep. 2017 Aug 30;7:10039. doi: 10.1038/s41598-017-09968-7 (PMC5577294; doi:10.1038/s41598-017-09968-7)
Supplement: Supplementary file 1 — Supplementary Information [file 41598_2017_9968_MOESM1_ESM.doc]

**Supplementary Information**

**“Plasma biomarker proteins for detection of human growth hormone administration in athletes”**

Sock-Hwee Tan^1,a^, Albert Lee^1,b^, Dana Pascovici^1^, Natasha Care^1^, Vita Birzniece^2,c^, Ken Ho^2,d^, Mark P. Molloy^1^, and Alamgir Khan^1^*

^1^Australian Proteome Analysis Facility (APAF), Level 4, Building F7B, Research Park Drive, Macquarie University, Sydney NSW 2109 Australia.

^2^ Garvan Institute of Medical Research, NSW 2010, Australia

^a^ Cardiovascular Research Institute, Yong Loo Lin School of Medicine, National University of Singapore.

^b^ current address: Department of Biomedical Sciences, Faculty of Medicine and Health Sciences, Macquarie University, NSW 2109, Australia

^c^ current address: School of Medicine, Western Sydney University, NSW 2751, Australia

^d^ current address: Princess Alexandra Hospital, University of Queensland, Brisbane, QLD 4102, Australia

**HAPs removal from human plasma and reproducibility of depletion runs**

MARS-7 (Agilent Technologies) human immuno-affinity column was used to remove seven interfering high abundance proteins (HAPs) such as albumin, IgG, IgA, transferrin, haptoglobin, antitrypsin, and fibrinogen prior to 2-D DIGE gel and iTRAQ MS/MS analyses to increase the detectable dynamic range. Two MARS-7 columns were used for HAPs removal from 112 plasma samples in triplicate (one column for each Phase of analysis). We were interested to find out how the column ages and how it removes HAPs at the beginning and also after completion of all the samples. To investigate this, we have run a control plasma before the 1^st^ experimental run and also after completion of 180^th^ run in each Phase. Chromatograms of both runs (at the beginning and at the end) were overlaid with minor deviation demonstrating an excellent reproducibility of the HAPs removal throughout (Fig. S1A). The amount of proteins (HAPs) removed varied amongst individuals, ranging between 40-50% based on chromatogram areas (Fig. S1B). However, overall coefficient of variation (CV) of all the depletion runs within an individual was approximately 3.5%.


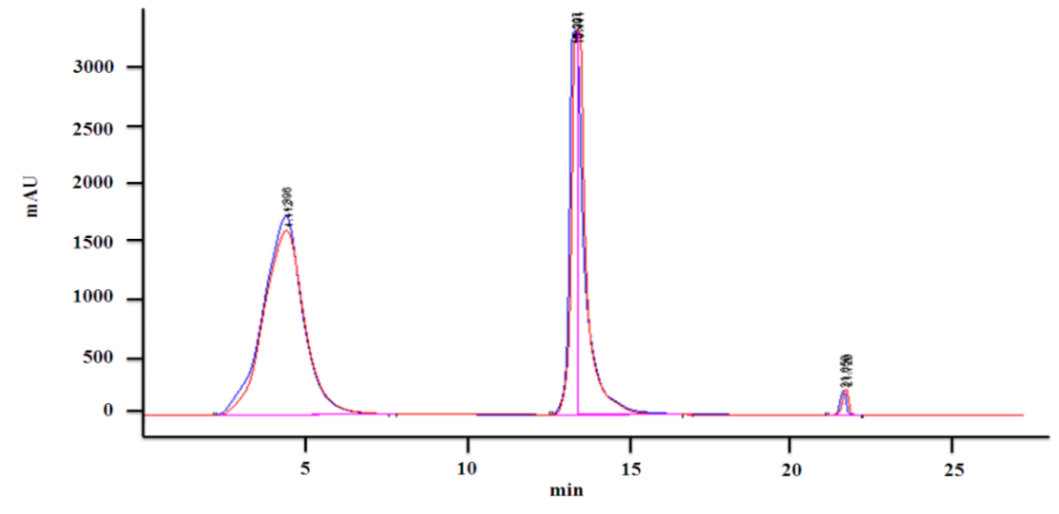


**A**


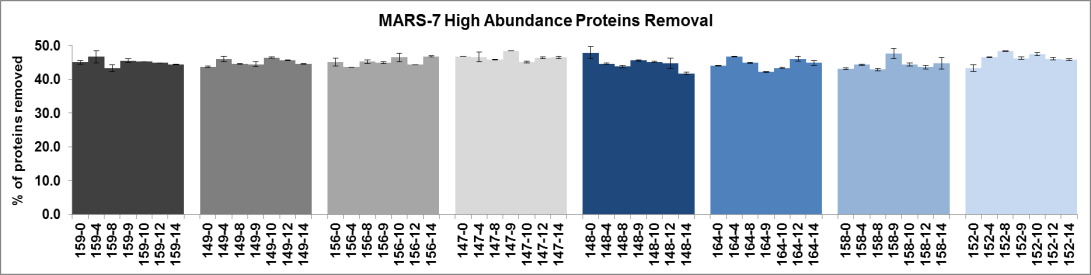


**B**

Figure S1. Overlaid chromatograms obtained from two separate runs (before the 1^st^ and after the 180^th^ run) of the healthy volunteer plasma samples onto the MARS-7 column (A); percent removal of the seven high abundance proteins from the plasma samples based on chromatogram areas (B). Each plasma sample was depleted in triplicate and error bar represented coefficient of variation (CV) between runs.

**2-D DIGE gel images (56 DIGE gels, 112 samples) produced and analyzed**

**
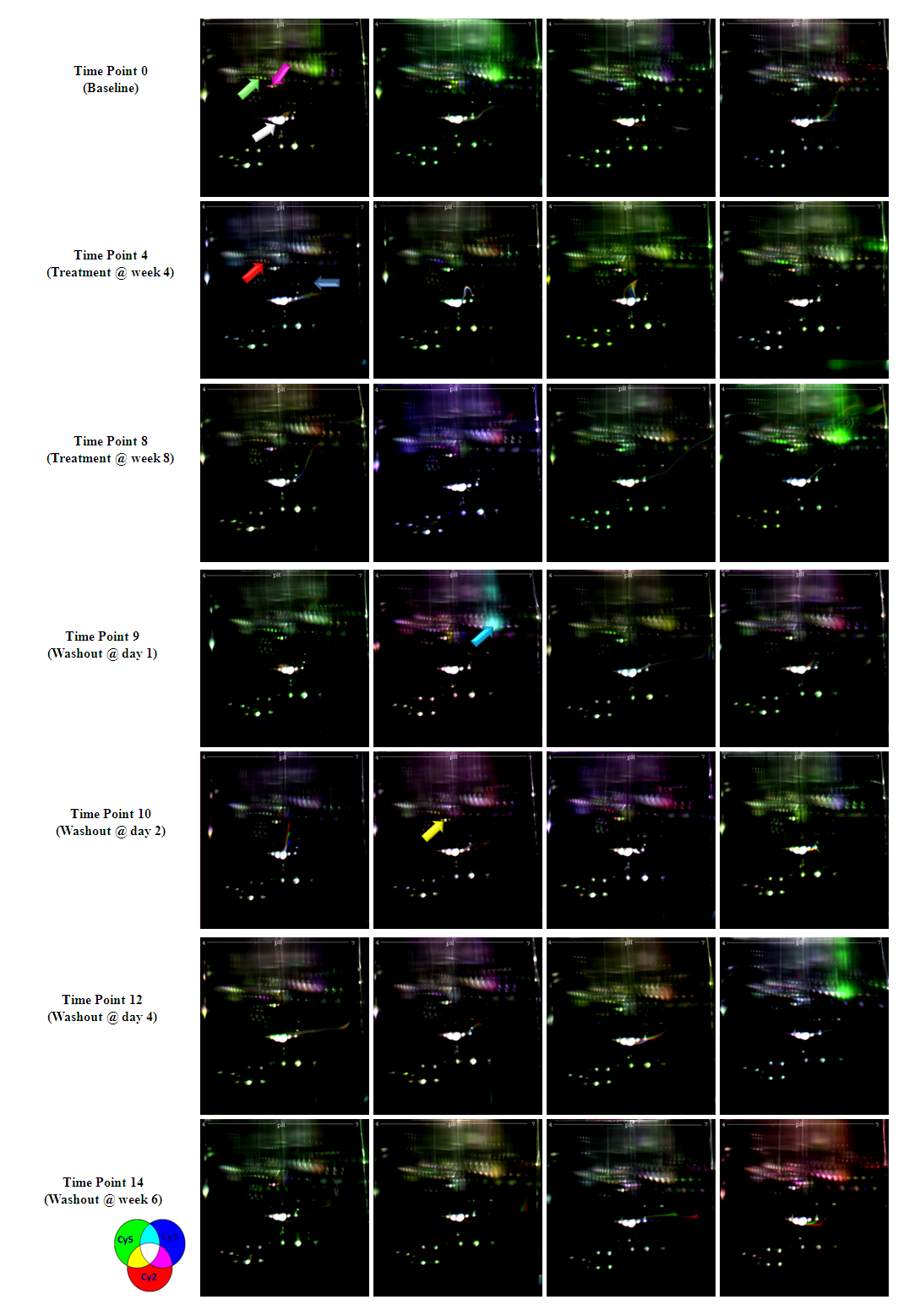
**

Figure S2A: Overlaid images of the 2D DIGE gels (Phase 1, 1-28 gels). Fifty-six samples were paired and separated on 28 gels. Each image represented 3 samples; placebo-treated, rGH-treated, and internal standard (pool of all 56 sampels). Sample pairing for each gel is indicated in Supplementary Table S2. Cy2: green, Cy3: orange, Cy5: red.


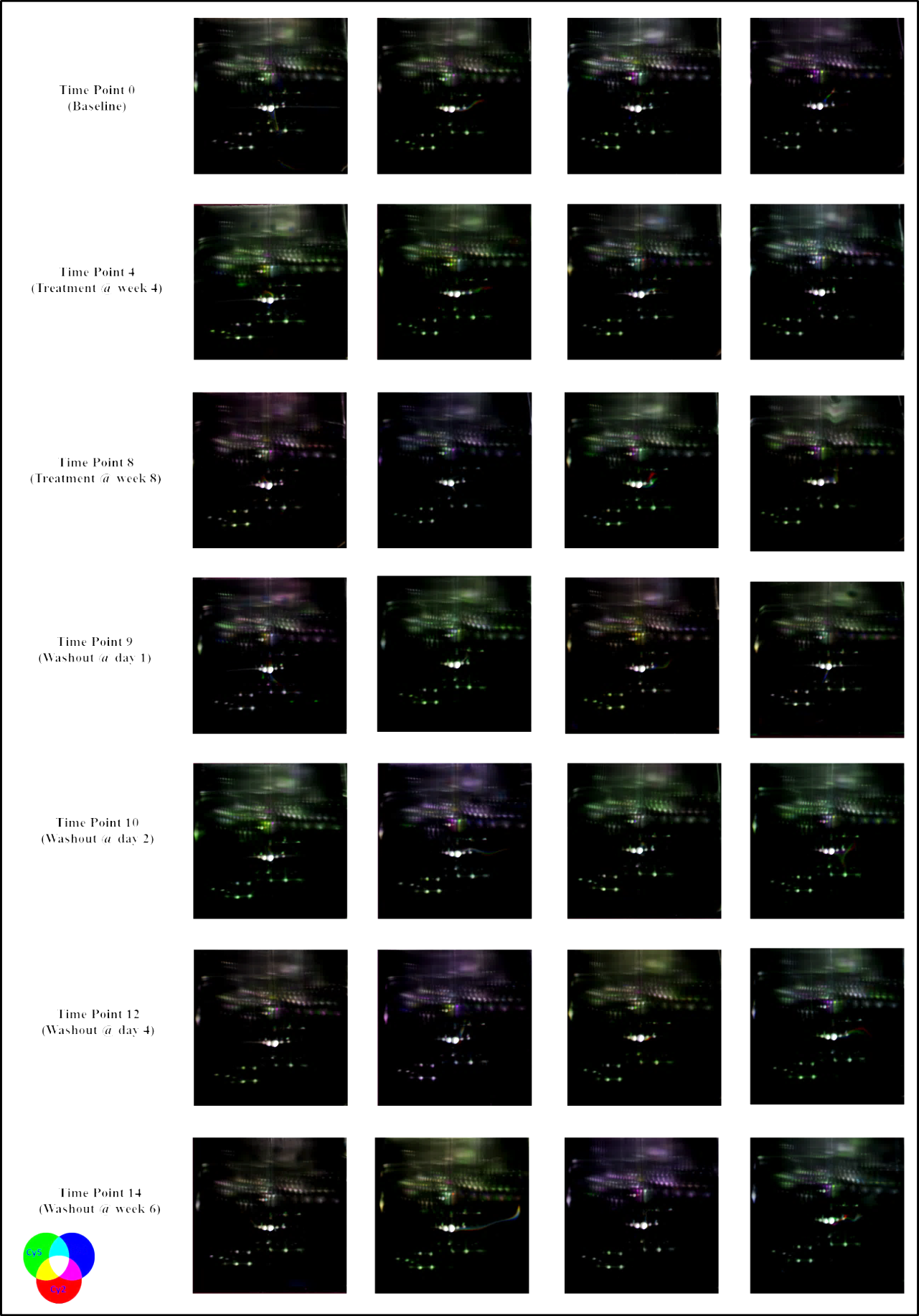


Figure S2B: Overlaid images of the 2D DIGE gels (Phase 2, 29-56 gels). Fifty-six samples were paired and separated on 28 gels. Each image represented 3 samples; placebo-treated, rGH-treated, and internal standard (pool of all 56 sampels). Sample pairing for each gel is indicated in Supplementary Table S2. Cy2: green, Cy3: orange, Cy5: red.

**Densitometric Analyses of 1-D Western blots of APOL1 and AHSG**

We have carried out 1-D Western blotting with samples from all the placebo-treated and rGH-treated subjects collected at all seven time points (baseline, treatment week 4, treatment week 8, washout day 1, washout day 2, washout day 4, and washout week 6) for both APOL1 and AHSG proteins. Densitometric analysis was performed on the detected bands on the 1-D Western blots and presented the difference between placebo-treated and rGH-treated across all the time points (Fig. S3). We have loaded equal amount of neat plasma proteins in each lane (20µg). Normalisation was carried out on the intensity of protein bands within each lane initially, and then normalised all measurements relative to the first lane.


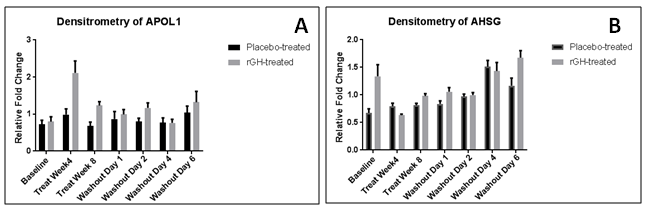


Figure S3. Densitometric analysis of APOL1 and AHSG from 1-D Western blots.

It is noteworthy that there is a difference in the baseline levels between placebo-treated and rGH-treated subjects for AHSG in Figure S3B. Two subjects from the rGH-treated cohort showed abnormally high AHSG levels (2.2 and 2.1-fold respectively) before administration which increased the averaged group AHSG level. These abnormally higher levels of AHSG in these two subjects could be attributed to inter-individual subject variation (age, sex, diet, exercise, etc.).  If both subjects were removed as outliers for densitometric analysis, the relative fold change of AHSG for the rGH-treated group would average to ~1.06-fold (no significance difference to placebo at baseline).  To keep the densitometric analysis consistent, we have included both subjects in the analysis.

**Quality of long term stored plasma used in this work vs freshly collected plasma**


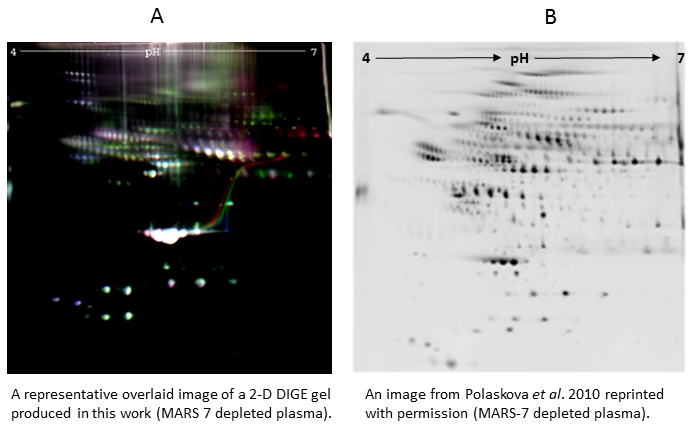


**Figure S4.** 2-D gel image comparison: a representative image produced in this work (**A**) vs a reported image (**B**). Image in **B** was reprinted from Polaskova *et al*. with permission [*Polaskova, V., Kapur, A., Khan, A., Molloy, M.P., and Baker, M.S. (2010) High-abundance protein depletion: Comparison of methods for human plasma biomarker discovery. Electrophoresis 31, 471-482*]. Overall 2-D gel spot pattern in **A** (long term stored plasma) was similar to the freshly collected plasma in **B** (pooled plasma from several volunteers). Both images were produced after removing the top seven high abundance proteins using MARS-7 columns. **A** is the CyDye labelled overlaid DIGE gel image and **B** is the Flamingo Pink stained gel.

The plasma samples analysed in this work were collected for another study *[r*e*f 4 in the main text]*, stored at -80^o^C throughout and each aliquot of plasma was thawed once only for analyses. Previous studies have shown no significant alterations to plasma integrity and quality after four years of storage observed by MALDI MS analysis at peptide level *[Mitchell, B.L., Yasui, Y., Li, C.I., Fitzpatrick, A.L., and Lampe, P.D. (2005) Impact of freeze-thaw cycles and storage time on plasma samples used in mass spectrometry based biomarker discovery projects. Cancer Infor****m.1****, 98-1*04]. Additionally a single freeze-thaw cycle, storage of serum at 4^o^C for one day and at -20^o^C for up to three months had no significant effect on two GH plasma biomarkers IGF-I or P-III-NP; concentration measured by immune-assays *[Guha, N., Erotokritou-Mulligan, I., Bartlett, C., Cowan, D.A., Bassett, E.E., Stow, M., Sönksen, P.H., and Holt, R.I. (2012) The effects of a freeze-thaw cycle and pre-analytical storage temperature on the stability of insulin-like growth factor-I and pro-collagen type III N-terminal propeptide concentrations: Implications for the detection of growth hormone misuse in athletes. Drug Test Anal. 4, 455-4*59]. However, similar storage effect data is not available where plasma was analysed by 2-D gel electrophoresis at protein level. As a long-term storage period has no major effect on plasma at the peptide level, it would not have an adverse impact on the protein level either. In this regard, a representative 2-D DIGE gel image of this work (overlaid image composed of 2 plasma samples and an internal standard) could be compared with a reported 2-D gel image where freshly collected plasma was used which was also produced after HAP removal using MARS column as used in this work (Fig. S4). Similar plasma protein profiles were observed in the 2-D gel images of freshly collected plasma and freeze-thawed plasma of two different healthy volunteers, which further highlights that plasma retains its integrity if stored at -80^o^C for extended periods of time.

**Principal Component Analysis (PCA) and selection of potential biomarker protein spots from the 2-D DIGE gels**


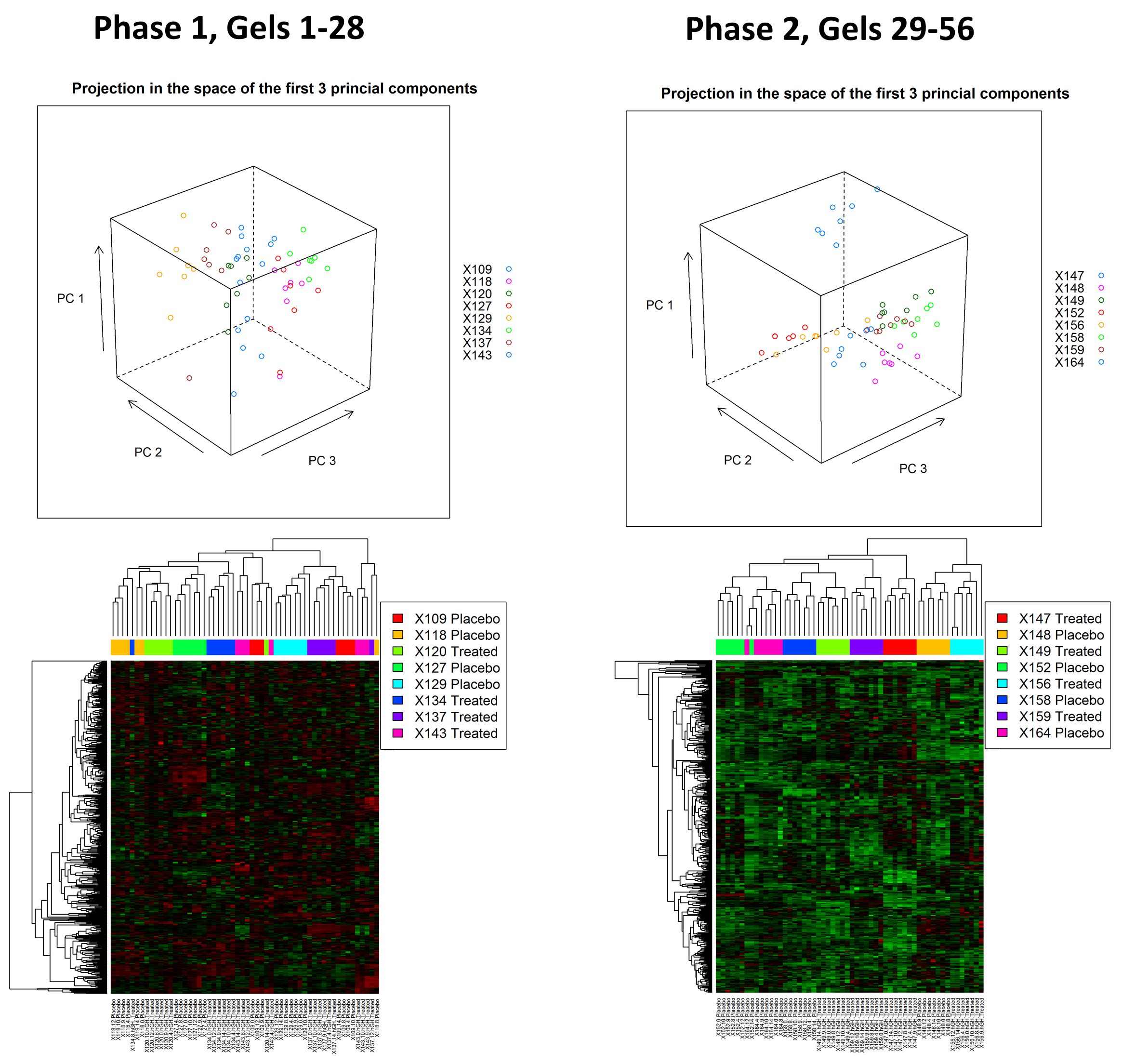


**Figure S5.** Principal Component Analysis (PCA) was carried out on the log-transformed spot data as well as hierarchical clustering using correlation based distance and complete linkage of the first half of the samples in phase 1 (1 to 56 samples of 4 placebo-treated and 4 rGH-treated over baseline, treatment weeks 4 and 8, washout day 1, day 2, day 4 and wk 6) and the second half of the samples in phase 2 (remaining 56 to 112 samples). The PCA shows samples from the same patient cluster together as expected.

**Preliminary selection criteria of potential candidate biomarker protein spots on DIGE gels**

Using the spot intensity data and a mixed effects model with treatment and time as fixed effects and patient as random effect, candidate spots were selected based on the following criteria:

**a.**       The mixed model p-value was found to be less than 0.05 for either the time effect, treatment effect or their interaction AND

**b.**      The relative change in the rGH-treated samples along the time course was greater than 50% (explained in detail below) OR the relative change between rGH-treated and placebo-treated samples was greater than 50% AND

**c.**       The relative change in the placebo-treated samples along the time course was less than 30%.

Criterion **a**. is a standard requirement, whilst **b**. and **c**. aimed to capture spots that showed temporal changes in the rGH-treated samples or between rGH-treated and placebo-treated along the time course, but no significant temporal changes in the placebo-treated samples.

The relative changes were calculated as follows:

**i)** The data for each spot was first averaged by sample and time point (obtaining averages for placebo-treated 0 (baseline), treatment wks 4 and 8, wash day 1, day 2, day 4 and wk 6; and rGH-treated 0 (baseline), treatment wks 4 and 8, wash day 1, day 2, day 4 and wk 6), and then relative changes in rGH-treated samples were calculated as:

- **MaxDiffrGH-treated = (Max rGH-treated – Min rGH-treated)/(Min rGH-treated)** - hence the requirement of relative change > 0.5 meant that the maximum average rGH-treated sample along the time course was greater than 1.5 the minimum average treated sample.

- **MaxDiffPlacebo-treated** (the relative change between placebo-treated samples) was calculated in the same way as using rGH-treated samples.

- **MaxDiffrGH-treatedPlacebo** is the relative change between rGH-treated and placebo-treated, and was calculated as the maximum of the difference between the rGH-treated and placebo-treated samples (the time course), compared to the smallest sample, e.g. Abs (rGH-treated at T0 – placebo-treated at T0)/min (rGH-treated at T0, Placebo-treated at T0).

Table S1: Differentially expressed 2-D DIGE gel protein spots identified by MALDI MS/MS analysis.

| **2-D Spot ID** | **SwissProt Entry Name** | **Protein Name** | **MASCOT score** | **Matched**  **peptides** | **Seq. Cov.** | **Mass (Da)** | **pI** |
| --- | --- | --- | --- | --- | --- | --- | --- |
| **Phase 1** | | | | | | | |
| **628** | [CO3_HUMAN](../../../../C:%5CDocuments%20and%20Settings%5Cstan%5CLocal%20Settings%5CTemporary%20Internet%20Files%5CContent.MSO%5C294AB5D6.xlsx#RANGE!Hit1) | Complement C3 | 448 | 48 | 36% | 187030 | 6.02 |
| **666** | CO3_HUMAN | Complement C3 | 368 | 32 | 26% | 187030 | 6.02 |
| **832** | [A1AT_HUMAN](http://apaf-sv-mascot/mascot/cgi/protein_view.pl?file=..%2Fdata%2F20130515%2FF082441.dat&hit=1) | Alpha-1-antitrypsin | 296 | 32 | 63% | 46707 | 5.37 |
| **878** | [A2GL_HUMAN](../../../../C:%5CDocuments%20and%20Settings%5Cstan%5CLocal%20Settings%5CTemporary%20Internet%20Files%5CContent.MSO%5C56BE98C3.xlsx#Sheet2!Hit1) | Leucine-rich alpha-2-glycoprotein | 165 | 7 | 20% | 38154 | 6.45 |
| **889** | APOL1_HUMAN | Apolipoprotein L1 | 64 | 8 | 19% | 43947 | 5.6 |
| **1609** | [A2GL_HUMAN](http://apaf-sv-mascot/mascot/cgi/protein_view.pl?file=..%2Fdata%2F20130515%2FF082472.dat&hit=1) | Leucine-rich alpha-2-glycoprotein | 67 | 7 | 19% | 38154 | 6.45 |
| **1614** | [A2GL_HUMAN](http://apaf-sv-mascot/mascot/cgi/protein_view.pl?file=..%2Fdata%2F20130808%2FF083549.dat&hit=1) | Leucine-rich alpha-2-glycoprotein | 165 | 8 | 20% | 38154 | 6.45 |
| **1682** | [A1AT_HUMAN](http://apaf-sv-mascot/mascot/cgi/protein_view.pl?file=..%2Fdata%2F20130515%2FF082440.dat&hit=1) | Alpha-1-antitrypsin | 334 | 35 | 65% | 46707 | 5.37 |
| **1691** | [KNG1_HUMAN](http://apaf-sv-mascot/mascot/cgi/protein_view.pl?file=..%2Fdata%2F20130808%2FF083547.dat&hit=1) | Kininogen-1 | 412 | 16 | 28% | 71912 | 6.34 |
| **1815** | [VTNC_HUMAN](http://apaf-sv-mascot/mascot/cgi/protein_view.pl?file=..%2Fdata%2F20130808%2FF083554.dat&hit=1) | Vitronectin | 156 | 14 | 26% | 54271 | 5.55 |
|  | [ANGT_HUMAN](http://apaf-sv-mascot/mascot/cgi/protein_view.pl?file=..%2Fdata%2F20130808%2FF083554.dat&hit=2) | Angiotensinogen | 119 | 10 | 25% | 53121 | 5.87 |
|  | [KNG1_HUMAN](http://apaf-sv-mascot/mascot/cgi/protein_view.pl?file=..%2Fdata%2F20130808%2FF083554.dat&hit=3) | Kininogen-1 | 148 | 19 | 31% | 71912 | 6.34 |
| **1821** | [VTNC_HUMAN](http://apaf-sv-mascot/mascot/cgi/protein_view.pl?file=..%2Fdata%2F20130515%2FF082408.dat&hit=1) | Vitronectin | 156 | 14 | 26% | 54271 | 5.55 |
| **862** | [A2GL_HUMAN](http://apaf-sv-mascot/mascot/cgi/protein_view.pl?file=..%2Fdata%2F20130515%2FF082470.dat&hit=1) | Leucine-rich alpha-2-glycoprotein | 153 | 9 | 28% | 38154 | 6.45 |
| **1260** | [SAA1_HUMAN](http://apaf-sv-mascot/mascot/cgi/protein_view.pl?file=..%2Fdata%2F20130808%2FF083569.dat&hit=1) | Serum amyloid A-1 protein | 56 | 3 | 34% | 13524 | 6.28 |
|  | [SAA2_HUMAN](http://apaf-sv-mascot/mascot/cgi/protein_view.pl?file=..%2Fdata%2F20130808%2FF083569.dat&hit=2) | Serum amyloid A-2 protein | 56 | 3 | 31% | 13519 | 9.2 |
| **1819** | [VTNC_HUMAN](http://apaf-sv-mascot/mascot/cgi/protein_view.pl?file=..%2Fdata%2F20130515%2FF082431.dat&hit=1) | Vitronectin | 170 | 16 | 33% | 54271 | 5.55 |
| **1921** | [FETUA_HUMAN](http://apaf-sv-mascot/mascot/cgi/protein_view.pl?file=..%2Fdata%2F20130808%2FF083555.dat&hit=2) | Alpha-2-HS-glycoprotein | 109 | 4 | 13% | 39300 | 5.43 |
| **694** | [CO3_HUMAN](../../../../C:%5CDocuments%20and%20Settings%5Cstan%5CLocal%20Settings%5CTemporary%20Internet%20Files%5CContent.MSO%5C294AB5D6.xlsx#RANGE!Hit1) | Complement C3 | 584 | 36 | 23% | 187030 | 6.02 |
| **838** | VTDB_HUMAN | Vitamin D-binding protein | 397 | 41 | 77% | 52929 | 5.4 |
| **1503** | APOA4_HUMAN | Apolipoprotein A-IV | 449 | 49 | 70% | 45371 | 5.82 |
| **1629** | [VTDB_HUMAN](../../../../C:%5CDocuments%20and%20Settings%5Cstan%5CLocal%20Settings%5CTemporary%20Internet%20Files%5CContent.MSO%5C56BE98C3.xlsx#Sheet2!Hit1) | Vitamin D-binding protein | 534 | 34 | 77% | 52929 | 5.4 |
| **2156** | [CO3_HUMAN](../../../../C:%5CDocuments%20and%20Settings%5Cstan%5CLocal%20Settings%5CTemporary%20Internet%20Files%5CContent.MSO%5C294AB5D6.xlsx#RANGE!Hit1) | Complement C3 | 507 | 40 | 28% | 187030 | 6.02 |
| **Phase 2** | | | | | | | |
| 246 | AACT_HUMAN | Alpha-1-antichymotrypsin | 438 | 19 | **45%** | 47621 | 5.33 |
| 252 | CO4A_HUMAN | Complement C4-A | 78 | 15 | **10%** | 192664 | 6.65 |
| 256 | CO4A_HUMAN | Complement C4-A | 89 | 19 | **13%** | 192664 | 6.65 |
| 257 | AACT_HUMAN | Alpha-1-antichymotrypsin | 65 | 9 | **26%** | 47621 | 5.33 |
| 275 | ITIH4_HUMAN | Inter-alpha-trypsin inhibitor heavy chain H4 | 144 | 17 | **20%** | 103293 | 6.51 |
| 290 | C1S_HUMAN | Complement C1s subcomponent | 42 | 4 | **6%** | 76635 | 4.86 |
| 522 | VTDB_HUMAN | Vitamin D-binding protein | 246 | 12 | **26%** | 52929 | 5.4 |
| 531 | FETUA_HUMAN | Alpha-2-HS-glycoprotein | 90 | 4 | **17%** | 39300 | 5.43 |
| 563 | APOL1_HUMAN | Apolipoprotein L1 | 108 | 7 | **19%** | 43947 | 5.6 |
| 564 | APOL1_HUMAN | Apolipoprotein L1 | 232 | 16 | **31%** | 43947 | 5.6 |
| 565 | APOL1_HUMAN | Apolipoprotein L1 | 126 | 7 | **17%** | 43947 | 5.6 |
| 567 | APOL1_HUMAN | Apolipoprotein L1 | 289 | 16 | **32%** | 43947 | 5.6 |
| 618 | APOE_HUMAN | Apolipoprotein E | 478 | 29 | **56%** | 36132 | 5.65 |
| 629 | APOE_HUMAN | Apolipoprotein E | 601 | 29 | **64%** | 36132 | 5.65 |
| 1021 | FETUA_HUMAN | Alpha-2-HS-glycoprotein | 145 | 6 | **18%** | 39300 | 5.43 |
| 1022 | VTNC_HUMAN | Vitronectin | 170 | 16 | **33%** | 54271 | 5.55 |
| 1046 | ANT3_HUMAN | Antithrombin-III | 324 | 16 | **33%** | 52569 | 6.32 |
|  | KNG1_HUMAN | Kininogen-1 | 171 | 17 | **26%** | 71912 | 6.34 |
| 1062 | FETUA_HUMAN | Alpha-2-HS-glycoprotein | 121 | 3 | **11%** | 39300 | 5.43 |
| 1069 | APOH_HUMAN | Beta-2-glycoprotein 1 | 86 | 4 | **12%** | 38273 | 8.34 |

A total of 20 protein spots (corresponded to 12 unique proteins) in phase 1 and 19 protein spots (corresponded to 13 unique proteins) in phase 2 were identified.

**Labelling of proteins with CyDyes for running 2-D DIGE gels**

Two plasma samples were simultaneously separated on a single 2D DIGE gel. Hence, 56 plasma samples (in each Phase) were paired into 28 groups and ran 28 DIGE gels. Equal amount of internal standard (pool of 56 samples labelled with Cy2) was added in all the gels. Each gel contained a pair of placebo-treated and rGH-treated sample collected at the same time-point as shown in Table S2 (data shown for phase 1 only; phase 2 labelling and pairing were the same as for phase 1) and an internal standard. Proteins were labelled with CyDyes according to the instructions provided by manufacturer with the exception of 400pmol CyDye dye was used to label 100µg of proteins instead of recommended 50µg. Although each gel composed of rHG-treated and placebo-treated samples from the same time point, for image analysis all 84 gel images in each phase were overlaid, spot edited, spot volumes were estimated and compared across all the images irrespective of time points i.e. all samples were comparable to all samples.

**Table S2. Paring of CyDye labelled plasma samples for running DIGE gels.**

| **Gel** | **Placebo-treated** | **Dye** | **rGH-treated** | **Dye** | **Gel** | **Placebo- treated** | **Dye** | **rGH-treated** | **Dye** |
| --- | --- | --- | --- | --- | --- | --- | --- | --- | --- |
| **Gel 1** | S1 | Cy3 | S1 | Cy5 | **Gel 15** | S15 | Cy3 | S15 | Cy5 |
| **Gel 2** | S2 | Cy5 | S2 | Cy3 | **Gel 16** | S16 | Cy5 | S16 | Cy3 |
| **Gel 3** | S3 | Cy3 | S3 | Cy5 | **Gel 17** | S17 | Cy3 | S17 | Cy5 |
| **Gel 4** | S4 | Cy5 | S4 | Cy3 | **Gel 18** | S18 | Cy5 | S18 | Cy3 |
| **Gel 5** | S5 | Cy3 | S5 | Cy5 | **Gel 19** | S19 | Cy3 | S19 | Cy5 |
| **Gel 6** | S6 | Cy5 | S6 | Cy3 | **Gel 20** | S20 | Cy5 | S20 | Cy3 |
| **Gel 7** | S7 | Cy3 | S7 | Cy5 | **Gel 21** | S21 | Cy3 | S21 | Cy5 |
| **Gel 8** | S8 | Cy5 | S8 | Cy3 | **Gel 22** | S22 | Cy5 | S22 | Cy3 |
| **Gel 9** | S9 | Cy3 | S9 | Cy5 | **Gel 23** | S23 | Cy3 | S23 | Cy5 |
| **Gel 10** | S10 | Cy5 | S10 | Cy3 | **Gel 24** | S24 | Cy5 | S24 | Cy3 |
| **Gel 11** | S11 | Cy3 | S11 | Cy5 | **Gel 25** | S25 | Cy3 | S25 | Cy5 |
| **Gel 12** | S12 | Cy5 | S12 | Cy3 | **Gel 26** | S26 | Cy5 | S26 | Cy3 |
| **Gel 13** | S13 | Cy3 | S13 | Cy5 | **Gel 27** | S27 | Cy3 | S27 | Cy5 |
| **Gel 14** | S14 | Cy5 | S14 | Cy3 | **Gel 28** | S28 | Cy5 | S28 | Cy3 |


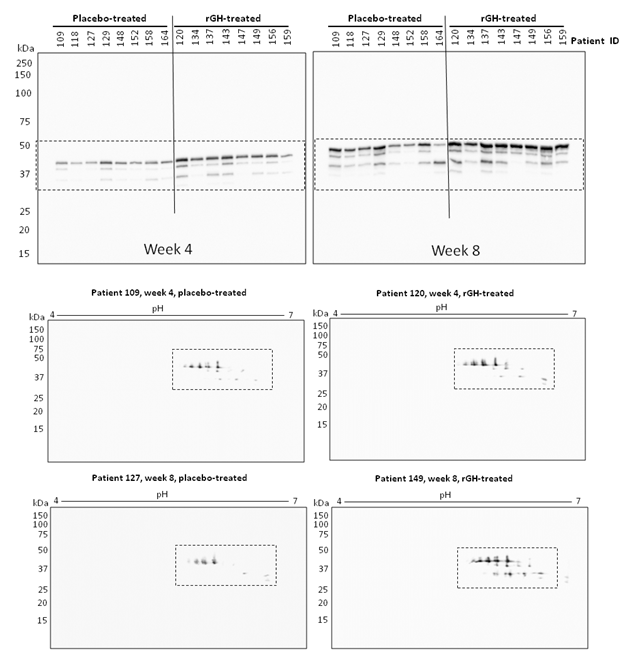


**Figure S6A**. Un-cropped full length Western blot images of APOL1. These images were cropped in the dotted boxed areas and shown in the Figure 6A of the main text.


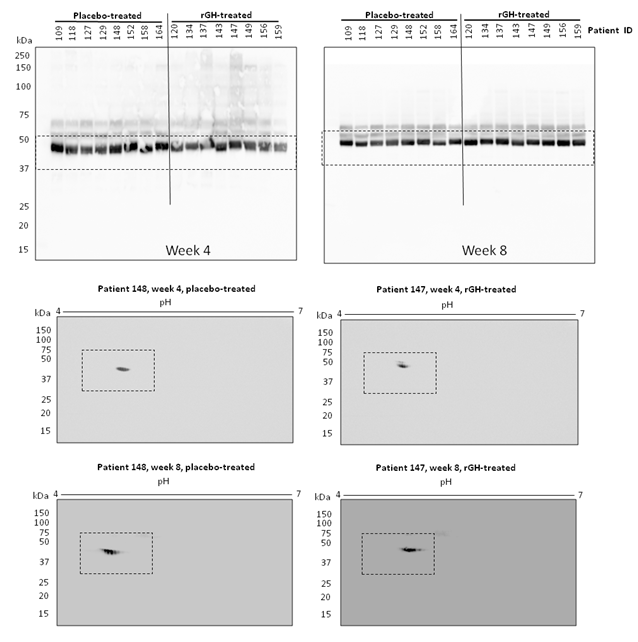


**Figure S6B**. Un-cropped full length Western blot images of AHSG. These images were cropped in the dotted boxed areas and shown in the Figure 6B of the main text. In the 1-D Western blots, additional bands were observed which may be related to glycosylation and phosphorylation of AHSG.
